# Medical students’ self-efficacy in problem-based learning and its relationship with self-regulated learning

**DOI:** 10.3402/meo.v21.30049

**Published:** 2016-03-16

**Authors:** Meral Demirören, Sevgi Turan, Derya Öztuna

**Affiliations:** 1 Department of Medical Education and Informatics, Ankara University School of Medicine, Dikimevi, Ankara, Turkey; 2 Department of Medical Education and Informatics, Hacettepe University School of Medicine, Sihhiye, Ankara, Turkey; 3 Department of Biostatistics, Ankara University School of Medicine, Sihhiye, Ankara, Turkey

**Keywords:** self-regulated learning, problem-based learning, self-efficacy, medical students, medical education

## Abstract

**Background:**

Problem-based learning (PBL) is most commonly used in medical education to enhance self-regulated learning (SRL) skills. Self-efficacy beliefs affect students’ motivation through self-regulatory processes. The purpose of this study is to examine the relationship between medical students’ self-reported SRL skills and their self-efficacy in PBL.

**Methods:**

A cross-sectional study was conducted with second (286; 83.1%) and third (275; 80.2%) year students at the Ankara University School of Medicine. The SRL perception (SRLP) scale and self-efficacy for problem-based learning (SPBL) scale were used in the study.

**Results:**

The SRLP subscales were positively correlated with the SPBL subscales. There was a weak but meaningful correlation between the subscales of SRLP (with the exception of the lack of self-directedness scale) and the subscales of SPBL and the students’ views on benefiting from PBL. The female students’ mean score was higher for the ‘planning and goal setting’ subscale of SRLP (*p=*0.017), and the second-year students’ mean score was higher than that of the third-year students for the ‘lack of self-directedness’ subscale of SRLP (*p=*0.001) with small effect sizes (Cohen's *d* is 0.17 and 0.27). There was no statistically significant difference between the year and subscales of SPBL. With regard to gender, the female students had higher scores than the male students on the ‘responsibility’ subscale of SPBL (*p=*0.003; Cohen's *d*=0.26).

**Conclusions:**

The study showed that medical students used SRL skills and believed in their ability to learn effectively in the PBL context and demonstrated the relationship between SRL skills and self-efficacy beliefs. Monitoring students’ development in these skills and giving them feedback could be beneficial for the cognitive achievement of students with learning difficulties and insufficient study skills. Further studies need to be undertaken to investigate issues such as the curriculum, learning environment, individual differences, and how these can affect the SRL process.

Physicians are required to have cognitive abilities that include problem-solving and decision-making skills, and sound clinical judgment. They also have a societal obligation to maintain their knowledge and skills by engaging in lifelong learning (1). Many physicians’ organizations have identified learning as a lifelong activity and have accepted the need to become a self-directed learner as an essential competency, recommending that these competencies should be improved and evaluated throughout the education of physicians (1–3). Correspondingly, medical educators have sought to ensure that students are well equipped with the necessary self-regulated learning (SRL) skills to cope with the continued exponential growth in medical knowledge. In medical education, problem-based learning (PBL) has become the preferred instructional methodology and curriculum approach to enhance SRL skills.

PBL aims to develop effective self-directedness (4). PBL helps students to develop effective problem-solving skills and to become active participants in their own learning by enabling them to construct knowledge (5, 6). In PBL, students learn content, strategies, and develop self-directed learning skills by collaboratively solving problems, reflecting on their experiences, and engaging in self-directed inquiry (7–10). Importantly, students are encouraged to take responsibility for their own learning through SRL (4, 11).

Self-regulation has been defined as the ability to self-regulate thoughts, feelings, and actions for attaining academic goals (12). Self-regulated learners effectively set goals, plan and use strategies to achieve their goals, manage their resources, and monitor and evaluate their progress at various stages of the learning process (13). In SRL, students participate in motivational, behavioral, and metacognitive aspects of their own learning (13). PBL articles use the term *self-directed learning* synonymously with *self-regulated learning* (i.e., SRL) in the educational psychology literature. We preferred to use the term SRL in this article because students in undergraduate education might not fulfill the condition of being fully independent as advocated by self-directed learning theorists.

Most research provides evidence that PBL fosters SRL (2, 9, 11, 14–19). When Schmidt et al. (20) asked medical graduates to rate their professional competencies, those from schools with PBL scored higher in interpersonal skills; had better competencies in problem solving, self-directed learning, and information gathering; and had better task-supporting skills such as the ability to work and plan efficiently. PBL students reported more engagement in SRL activities than those who qualified from the traditional program (18), and they were also more motivated to learn (2), had higher levels of intrinsic goal orientation and task value (17), and used more elaboration strategies, critical thinking, and metacognition (17). Thus, it was concluded that when students are responsible for their own learning, they acquire autonomous learning skills that are essential for lifelong learning (11). However, while most studies have reported positive results, some have reported negative results for PBL in improving SRL skills (21).

Self-regulatory skills are of little value if students do not motivate themselves to use them. One of the most studied self-motivational beliefs is self-efficacy, which refers to an individual's beliefs about his or her capabilities to learn or perform behavior at a defined level (22). Furthermore, self-efficacy beliefs are hypothesized to be mediators of behavioral change (13–23) and develop from four sources: direct experiences, vicarious experiences from observing peers, persuasion by others, and personal physiological reactions (22).

Students with high levels of self-efficacy are more willing to take on challenging tasks (24). When facing a difficult learning task, a student with high self-efficacy is more likely to participate actively, work harder, remain more problem focused, and persevere for a longer time than a student with low self-efficacy, who is more likely to become frustrated and give up (25). Due to the students’ responsibility for SRL in PBL, this form of education requires drastic changes in the roles of students and tutors. However, many studies have shown that students do not adapt easily to the change, and that self-efficacy beliefs can be both indicators of change during instructional interventions and indicators of initial individual differences (24). Therefore, self-efficacy is an important variable that can be used to predict behavioral change in students’ roles.

Gender differences in SRL skills have been investigated in numerous studies. The studies have been reported that women are more likely to use specific learning strategies (26–30) and goal structure (26–31). Pajares (2002) reported that females displayed more goal setting and planning strategies, and that they kept records and structured their environment for optimal learning more frequently (32). The relationship between self-efficacy and gender has also been examined in wider general education. However, the context of the self-efficacy should not be ignored when these studies are reviewed. Gender differences and confidence have been studied widely in mathematics, science, language, and arts, with divergent gender differences in self-efficacy depending on the subject. However, gender differences in self-efficacy in PBL have not been considered in any studies to date. Mostly, gender differences are reported in academic self-efficacy and self-efficacy for employing self-regulatory strategies. It was indicated that females express greater self-efficacy in self-regulation and greater confidence in their ability to use specific learning strategies. These strategies include finishing homework assignments on time, studying when there are other things to do, remembering information presented in class and textbooks, and participating in class discussions (33–35). Although girls are perceived to be more responsible about their study, gender differences are determined by many factors, including cultural influences, which need to be investigated in the context of PBL.

The medical curriculum aims to increase students’ SRL skills and their belief in their ability to learn. Van den Hurk et al. (36) examined the planning aspect of SRL and showed that first-year students are uncertain about what literature should be studied and confine themselves to the specified content, whereas in later years, they study more according to their own learning needs and interests. In parallel with Van den Hurk et al. (36), we also tried to investigate the progress of students in developing SRL skills, and their beliefs in their ability to learn effectively in the PBL context. However, self-efficacy beliefs affect the level of motivation through self-regulatory processes (32, 37), and Bandura (38) stated that human motivation and behavior influence each other reciprocally. Therefore, increasing students’ self-efficacy beliefs should improve their motivation and SRL skills, and vice versa. This suggests that self-efficacy beliefs can be used to predict and promote medical students’ learning skills and motivation. Indeed, Kek and Huijser (39) argue that self-efficacy beliefs affect the improvement of SRL skills, and Papinczak et al. (25) reported a significant association between high self-efficacy and a deeper learning approach. However, they also found that students lose self-efficacy and move away from deep strategic learning approaches toward more surface approaches during the first year of medical studies. In this context, the structure of medical curricula is crucial. It is important to know how students with different levels of self-regulated strategies participate and perform in PBL, and how this is related to their self-efficacy beliefs. To date, though, there has been relatively little empirical research on SRL skills and self-efficacy beliefs of students with regard to PBL in medical education.

Numerous studies have been performed to investigate the relationship between SRL and self-efficacy. However, almost of these were not performed in medical contexts. Moreover, our literature search did not reveal any published study in the PBL setting. With these considerations in mind, we aimed to investigate the relationship between medical students’ SRL skills and self-efficacy beliefs as they relate to PBL. We used the SRL perception (SRLP) and self-efficacy for PBL (SPBL) scales to test the hypothesis that levels of self-directed learning and self-efficacy belief would increase concomitantly among students. The following research questions were posed:

1. What are the SRL skills and self-efficacy of medical students in PBL?
2. Are there any differences in the SRL skills and self-efficacy of medical students in PBL between different genders and years of study?
3. Is there a relationship between medical students’ views on the benefit of PBL and their self-reported SRL skills and self-efficacy in PBL?

## Methods

### Setting

This study was conducted at Ankara University School of Medicine (AUSM) in Turkey in the academic year 2013–14. At this medical school, a systematic and integrated program is being applied that allows for problem-, community-, and competency-based learning. The duration of AUSM's medical education program is 6 years, and the program has been structured as preclinical (years 1–3), clinical (years 4–5), and an internship (year 6).

The curriculum in the preclinical years consists of multidisciplinary learning modules, while in the clinical years, the program is predominantly structured into discipline-based clerkships. Each year consists of four modules that last 8 weeks each. Modules are structured on the basis of organ systems and consist of lectures (37% of both the second and third year), PBL sessions (5% of both the second and third year), laboratory practice (19% of the second year and 5% of the third year), clinical and communication skills practices (3% of the second year and 5% of the third year), evidence-based medicine (6% of the second year and 5% of the third year), and community medicine (10% of the third year). Although the proportion of small group sessions, such as PBL, is small, the curriculum program is designed to allow for extensive individual study (30% of the second year and 35% of the third year), which allows students to study independently and enhance their learning.

PBL is included in the second and third years, and each module includes two PBL scenarios. With PBL, in addition to teaching basic science in the clinical context, the course aims to ensure a multidimensional integration that covers behavioral, social, ethical, and biopsychosocial approaches to the patient. At the beginning of the second year, students are taught the roles of learners and tutors, the process, and the assessment process of PBL using interactive teaching techniques in small groups. The PBL tutors also attend a 3-day PBL tutoring course. The PBL tutors of the second-year students consist primarily of the basic science faculty, while the third-year PBL tutors are primarily from the clinical science faculty.

In PBL, groups of 11 students work together with a tutor. Each PBL scenario is processed in two or three sessions of 2 h each over 2 weeks. Between the sessions, students are expected to study independently toward their learning objectives, as defined in the previous session.

### Participants

This cross-sectional study was carried out with second- and third-year students. In the academic year 2013–14 at AUSM, there were 344 and 343 students in the second and third year, respectively. The students were informed about the study and asked to give their consent. Of the total sample, 286 (83.1%) second-year students and 275 (80.2%) third-year students participated in the study (total, 561 students). In total, 56% of the participants were female (316 females, 244 males), and the participation rate was 81.7%. The second- and third-year students completed both assessment scales at the end of the last PBL session in the autumn semester. Students who did not attend the PBL session did not participate in the study.

### Instruments

The SRLP scale was used to determine students’ self-reported use of SRL skills, and the SPBL scale was used to determine students’ self-reported motivation. The students were also asked about their views on benefiting from PBL, using a 10-point Likert scale.

#### Self-regulated learning perception scale

SRLP, a self-report scale developed by Turan (40), is not only used to determine students’ use of SRL strategies and goal settings, but also includes a motivation component to determine students’ focus on learning. The scale contains 41 items and 4 subscales. The items were rated on a 5-point Likert scale. The subscales were as follows:

- Motivation and action to learning: Students are willing to engage in learning and take action to learn.
- Planning and goal setting: Students formulate their goals and objectives and they plan their learning according to these objectives.
- Strategy use and assessment: Students choose and implement appropriate learning strategies to achieve their learning objectives and evaluate their learning outcomes.
- Lack of self-directedness: Students may have problems directing their learning process.

The Cronbach α coefficients for reliability have previously been reported as 0.88, 0.91, 0.83, and 0.76, respectively, for these 4 subscales (19, 40). In this study, the Cronbach α coefficients were calculated as 0.81, 0.89, 0.91, and 0.77, respectively.

#### Self-efficacy for problem-based learning scale

SPBL was developed by Onan et al. (41) to measure students’ beliefs about their ability to learn effectively through PBL. The scale items were based on the learning activities carried out by learners during the PBL tutorial. The self-reported scale included 18 items in three subscales, rated on 5-point Likert scales. The subscales were as follows:

- Group interaction: Students work collaboratively and communicate effectively with their group mates and tutors.
- Problem solving: Students discover, analyze, discuss problems, and determine their learning deficit to solve the problem.
- Responsibility: Students take responsibility for their own learning, as well as that of their group mates.

Cronbach α coefficients for reliability have previously been reported as 0.87, 0.76, and 0.71, for these 3 subscales, respectively (41). In this study, the Cronbach α coefficients were calculated as 0.88, 0.81, and 0.75, respectively.

### Analysis

The mean scores and standard deviations were calculated. The relationship between the students’ SRLP and SPBL scores was analyzed using Pearson's correlation. The student's *t*-test was used to compare the scores according to gender and year. The effect size was evaluated with Cohen's *d*.

### Ethical consideration

Participation was voluntary and participants gave written informed consent. The Clinical Research Ethical Committee of Ankara University approved the study.

## Results

There were no statistically significant differences in gender for the SRLP subscales, with the exception of the ‘planning and goal setting’ scores for which the mean score of the female students was higher than that for male students (*p*=0.017). When student age compared; most students were 20 or 21 years old in the second year (78.5%) and 21 or 22 years old in the third year (82.6%). Statistical comparison of the students’ ages was not possible due to the close age range of the students. Instead, comparison was conducted according to the registered year of the student. The second-year students had higher scores than the third-year students in ‘lack of self-directedness’ (*p*=0.001). Cohen's *d* was 0.17 for the ‘planning and goal setting’ subscale and 0.27 for the ‘lack of self-directedness’ subscale, but there were no differences in the other subscale scores (Table 1).

There were no statistically significant differences between the registered year and the SPBL subscales. Female students had higher scores than male students for the ‘responsibility’ subscale (*p*=0.003; Cohen's *d=*0.26), but there were no differences in the other subscale scores (Table 2).

There was a weak but meaningful correlation between students’ views on benefiting from PBL, and the SPBL and SRLP subscales (with the exception of the ‘lack of self-directedness’ subscale of the SRLP) (Table 3).

**Table 1:** Students’ mean SRLP scores according to their characteristics

	Subscales of SRLP (mean±standard deviation)				
	
	Motivation and action to learning	Planning and goal setting	Strategy use and assessment	Lack of self-directedness	Total
Year					
Second year (*n=*286)	3.91±0.55	3.79±0.68	3.73±0.50	3.43±0.68	3.71±0.46
Third year (*n*=275)	3.89±0.52	3.79±0.62	3.72±0.52	3.25±0.67	3.66±0.47
t;p*	0.56;>0.05	−0.05;>0.05	0.17;>0.05	3.07;<0.001**	1.12;>0.05
Gender					
Female (*n*=316)	3.92±0.52	3.84±0.63	3.73±0.49	3.39±0.66	3.71±0.46
Male (*n=*244)	3.88±0.55	3.73±0.67	3.71±0.54	3.28±0.71	3.66±0.47
t;p*	0.76;>0.05	2.02;<0.017**	0.28;>0.05	1.76;>0.05	1.16;>0.05
Total (*n*=561)	3.90±0.54	3.79±0.65	3.72±0.51	3.34±0.68	

* *t* is student's *t*-test.

** Cohen's *d* for planning and goal setting was 0.17; for lack of self-directedness this was 0.27.

**Table 2:** Students’ mean of SPBL scores according to their characteristics

	Subscales of SPBL (mean±standard deviation)			
	
	Group interaction	Responsibility	Problem solving	Total
Year				
Second year (*n*=285)	3.88±0.54	4.023±0.566	3.89±0.54	3.92±0.50
Third Year (*n*=275)	3.91±0.58	4.035±0.640	3.88±0.58	3.92±0.56
t;p*	−0.37; > 0.05	0.05; > 0.05	0.59; > 0.05	0.04; > 0.05
Gender				
Female (*n*=316)	3.91±0.54	4.10±0.55	3.93±0.52	3.95±0.50
Male (*n=*244)	3.88±0.59	3.94±0.66	3.83±0.61	3.87±0.57
t;p*	0.58; > 0.05	2.96;<0.003**	1.81; > 0.05	1.71; > 0.05
Total (*n*=560)	3.89±0.56	4.03±0.60	3.89±0.56	

* *t* is student's *t*-test.

** Cohen's *d* for responsibility was 0.26.

**Table 3:** The relationship between SRLP and SPBL subscales and students’ views on benefiting from PBL

Scales	Benefiting from PBL
Subscales of SRLP	Correlation coefficients
Motivation and action to learning	0.222*
Planning and goal setting	0.190*
Strategy use and assessment	0.218*
Lack of self-directedness	0.060
Subscales of SPBL	
Group interaction	0.219*
Responsibility	0.239*
Problem solving	0.218*

* *p* < 0.01.

The relationship between the SRLP and SPBL subscales is shown in Table 4. The SRLP subscales were positively correlated with the SPBL subscales: when scores for students’ SRL skills increased, their self-efficacy levels also increased.

**Table 4:** The relationship between SRLP and SPBL subscales

	Correlation coefficients		
	
	Subscales of SPBL		
	
Subscales of SRLP	Group interaction	Responsibility	Problem solving
Motivation and action to learning	0.457*	0.450*	0.427*
Planning and goal setting	0.438*	0.409*	0.449*
Strategy use and assessment	0.492*	0.450*	0.501*
Lack of self-directedness	0.212*	0.214*	0.252*

* *p* < 0.01.

## Discussion

The need for lifelong learning requires schools to prepare learners to engage in SRL. Indeed, SRL skills are considered the first step toward taking control of professional lives and are equated with independent study, which contrasts with study strategies that rely on memorizing without necessarily understanding (42). All models of SRL share certain assumptions, one of which is that students construct their own meanings, goals, and strategies based on the availability of internal or external information. Another assumption is that students are capable of monitoring and managing aspects of their own cognition, motivation, behavior, and learning environment (43). Monitoring can be seen as the assessment of feedback information, and managing has to do with taking control of learning tasks and activities (11). Hence, we assessed students’ motivation and action with regard to learning, planning, and goal setting and the strategies and assessment skills used in relation to their SRL skills.

In this study, the mean self-regulatory and self-efficacy scores of students were higher than the possible mean score; that is, [maximum score (5)+minimum score (1)]/2 =3. The lowest mean score was 3.34 in the ‘lack of self-directedness’ subscale. The items of this scale were negative, but they were all inversed to calculate the SRLP scales’ score. Therefore, the higher scores indicated more self-regulated skills, which is consistent with the results of a previous study with medical students in Turkey (19), although those authors reported a lower subscale score (2.8). These results showed that the medical students who participated in this study used SRL skills and believed in their ability to learn effectively through PBL. In line with these results, Shokar et al. (44) investigated the self-directed learning readiness scores of third-year medical students and reported that the scores of students who participated in a PBL curriculum were significantly higher than those of general adult learners (44).

Many medical curricula contain small activities that help to develop SRL skills. Similar to Van den Hurk et al. (36), our expectation was that as the students progressed in their education, their use of SRL skills, and therefore their beliefs in their ability to learn effectively in the PBL context, would increase. This expectation was not, however, supported by the study findings. There was no statistically significant difference between years two and three and the self-efficacy subscales. The only SRLP subscale for which there was a relationship between years was ‘lack of self-directedness’ (*p*=0.001), with a small effect size (Cohen's *d*= 0.27); however, a negative relationship was evident for this subscale, with second-year students having higher scores than third-year students. Papinczak et al. (25) also found that medical students move away from deep strategic learning approaches and toward surface approaches during the first year of study. Despite this change, Papinczak et al. argue that SRL and deep learning approaches are closely linked. In this regard, the medical curriculum might somehow support the move from deep to surface learning, making the nature of the medical curriculum essential. These results showed that there is greater need for intervention to improve SRL skills throughout the curriculum. Indeed, the inclusion of learner-centered methods, such as PBL, is important for the development of SRL skills in the initial years of a curriculum, while SRL skills should be increasingly supported in later years by implementing learner-centered methods in a more integrated manner.

The characteristics of the learner, the learning environment, and the curriculum (objectives, activities, assessment, and so on) can influence the SRL process (6, 18, 45, 46). The curriculum is a mixture of individual components, thereby making it difficult to make links between specific aspects of the curriculum and student behavior (47). As a consequence, although most studies have confirmed that PBL improves SRL skills (2, 9, 11, 14–19, 48), there have been some mixed results regarding the effect of PBL on SRL. Shokar et al. (44) reported that the self-directed learning readiness scores of third-year medical students in a PBL curriculum were higher than those of adult learners. However, Lumma-Sellenthin (21) found that students’ self-regulation skills in a PBL curriculum were higher than those of students following a curriculum with mixed teaching approaches in one school, but that there was no difference in another school with a PBL curriculum.

The curriculum used at the AUSM in this study was a hybrid that comprised lectures and student-centered sessions (PBL, skills labs, evidence-based medicine sessions, and so on). Lee et al. (49) asserted that students in a hybrid curriculum tend to rely on lectures instead of the tutorials and other components of the curriculum. In the AUSM program, students study new learning objectives in each PBL session. Although the SRL skills of the students were not low, the structure of the curriculum and the presence of lectures in the curriculum at the AUSM might create limitations on the potential for improvement of SRL skills. Therefore, the effects of the curriculum should be examined in greater depth in future studies. In addition, this study was only cross-sectional in design, which meant that we could not monitor the students to measure their progress in SRL skills. Longitudinal studies should be designed to explore the progress of SRL skills in individual students over time.

Lumma-Sellenthin (21) also stated that students’ earlier work experiences could be a possible moderator of self-regulation skills. Students need more regulation when teacher-directed learning decreases in the curriculum, and less regulation when teacher-directed context increases (46, 50). The general consensus is that primary and secondary (K-12) education is largely teacher-centered (51), which is also the case in Turkey. Furthermore, students can only enroll in medical school after outperforming many other students in a national exam, creating fierce competition. This context can nurture strategic learning approaches and achievement motivation (52). In the present study, SRL and the self-efficacy levels of the students were evaluated in the context of PBL, and the results indicate that medical students who participated in the study used SRL skills and believed in their ability to learn effectively in the PBL context. However, it is important to remember that the background characteristics of students and the various other components of the curriculum can also affect the development of SRL skills. Due to the cross-sectional design of this study, it is difficult to explain how these factors affect the result.

In our study, the differences in the SRLP total scores between genders were not significant, consistent with a previous study in Turkey (19). Similarly, Reio and Davis (53) found no significant difference by gender in the self-directed learning readiness scores of high school, dental, and adult educational center students. Harvey et al. (54) also confirmed this result. Despite these findings, some studies have implied that there are in fact significant differences in the use of SRL strategies in favor of females (27–30). However, only the ‘planning and goal setting’ subscale was higher for females in this study.

Self-regulated learners plan their learning activities carefully before they initiate a specific task. The starting point is to analyze and determine the task and its features. Subsequently, goals are set and plans are made to apply tactics and strategies to complete the tasks. During these steps, self-regulated learners reflect on the steps that were taken, monitor their progress, and change their plans accordingly (6). The findings of the study showed that females perceive themselves as better at the SRL planning process. There were significant gender differences in the ‘planning and goal setting’ subscale scores of the SRLP (*p*=0.017) and the ‘responsibility’ subscale scores of SPBL (*p=*0.003) favoring female students. As mentioned in the *Introduction* section of this report, girls might have more responsibility in their study. Although the effect size was small, further qualitative study might give more detailed information about why and how gender differences emerge, such as the role of cultural influences.

The theories proposed to date indicate that there is a relationship between self-regulation and self-efficacy in PBL, with self-efficacy beliefs affecting the level of motivation through self-regulatory processes (26, 28). Indeed, it has been shown that higher levels of self-efficacy are correlated with the use of higher levels of learning strategy (24, 28, 39, 55–57). In other words, effective self-regulatory practices can result in stronger self-efficacy (57). However, most of these studies reported results in general educational settings, while we have investigated this relationship in a medical curriculum using PBL. As expected, we demonstrated positive correlations between the subscales of SRLP and SPBL and conclude that, when students’ self-directed learning skills scores increase, their self-efficacy levels also increase.

Understanding group dynamics is important for PBL. A prerequisite for effective group functioning is collaboration among students (58); if the group members do not consider their work and formulate the premises for the tutorials, the quality of the inquiries in the situation and the learning process will suffer. In a group that functions well, each individual student can use the group to develop his or her own learning process while contributing to common goals (59). Several studies have found that a person's preference for group learning and his or her ability to self-regulate his or her learning strategies are positively correlated (18, 60, 61). This study also showed that there is a positive relationship between group interaction and SRL.

Silen (59) investigated the factors that are important in the development of self-directedness in learning. According to Silen, students’ feelings of being in charge and having a genuine impact on the learning situations are crucial to their desire to take responsibility. Moreover, the feeling of being in charge is connected to understanding the demands of the learning context, as well as the experiences of managing and getting feedback (59). Studies have indicated that being part of the decision-making process helps students to develop their self-regulation and self-monitoring skills (50, 62, 63). Similarly, we found that responsibility was found related to the SRL subscales in this study.

The students rated PBL utilization as 7.4 out of 10, suggesting that the students thought PBL was beneficial. We expected that, if the students benefited from PBL, their self-regulated skills and self-efficacy beliefs would also be affected. Although this assumption was investigated using correlation analysis, we only showed a weak but meaningful correlation between students’ views on benefiting from PBL, and the SPBL and SRLP subscales (with the exception of the ‘lack of self-directedness’ subscale of the SRLP). As expected, when students perceived that they benefited from PBL, their self-efficacy beliefs and SRL skills improved.

## Conclusions

The findings of this study show that medical students enrolled in a hybrid curriculum with PBL use SRL skills and believe in their ability to learn effectively in the PBL context. Positive correlations seem to exist between SRL skills and self-efficacy beliefs, with evidence of a relationship between students’ positive views on benefiting from PBL and SRL skills and self-efficacy beliefs. However, there may not be a significant difference between years with regard to SRL skills and self-efficacy in PBL.

While implementing learner-centered methods, students may have difficulties in taking learning responsibility and carrying out team work. The results of this study indicate that responsibility for learning, teamwork skills, and self-efficacy are all related to the development of SRL skills. In this context, students might be expected to benefit from learning environments that support the development of their learning skills and self-efficacy. Helping students to prepare for PBL and similar learning methods at the beginning of their education is warranted to maximize their benefit from these techniques.

Another important consideration is the need to give individual counseling and support to students. Monitoring the development of SRL skills and giving students feedback could be beneficial, especially for students who are having difficulties with the learning approach or who have insufficient study skills. Screening and supporting students who have low self-efficacy and problems arranging their own learning may significantly contribute to their conquering of these problems. Counseling for learning should be provided throughout medical education. Thus, there will be an increasing responsibility to provide learning and learning skills as experience of the student increases in medical schools. By common consent, education must be a lifelong, continuous process. Given this, implementing student-centered learning methods in K-12 classes may improve students’ SRL skills and support undergraduate medical education. Ensuring that students are better prepared during high school should give them the SRL skills to cope with the rigors of the undergraduate course and to improve throughout their medical careers.

There were several limitations to this study, including its single-institution, cross-sectional design, and the fact that we did not attempt to show a cause and effect relationship. In addition, like all self-reported questionnaires, the scales used in the study have reliability and validity limitations. Despite this, the reliability analyses suggested that the scales had reasonable psychometric properties.

This study does provide direction for further research. We plan to investigate students’ skills and beliefs in a longitudinal study throughout medical education and to investigate the effect of tutors’ roles on acquiring SRL skills. In addition, a notable result of our study was that female students scored higher than male students in the ‘planning and goal setting’ and ‘responsibility’ subscales. Due to the limitations of the study design and the existence of conflicting literature, further studies are needed to clarify how the curriculum, the learning environment, and the presence of individual differences affect SRL.
